# Citrus Wastes as Source of Pectin and Bioactive Compounds Extracted via One-Pot Microwave Process: An In Situ Path to Modulated Property Control †

**DOI:** 10.3390/polym17050659

**Published:** 2025-02-28

**Authors:** Domenico Zannini, Martina Monteforte, Luca Gargiulo, Tiziana Marino, Giovanna Gomez d’Ayala, Gabriella Santagata, Giovanni Dal Poggetto

**Affiliations:** 1 Institute of Chemical Sciences and Technologies “G. Natta” (SCITEC), National Council of Research, Via De Marini 6, 16149 Genova, Italy; domenico.zannini@cnr.it; 2 Institute for Polymers, Composites and Biomaterials (IPCB), National Council of Research, Via C. Flegrei 34, 80078 Pozzuoli, Italy; martinamonteforte15@gmail.com (M.M.); lucagargiulo@cnr.it (L.G.); tiziana.marino@cnr.it (T.M.); giovanna.gomezdayala@cnr.it (G.G.d.); giovanni.dalpoggetto@cnr.it (G.D.P.)

**Keywords:** citrus pomace, microwave-assisted extraction, one-pot strategy, pectin, bioactive compounds, in situ property control

## Abstract

In this paper, citrus pomace was used as a source of pectin and polyphenols extracted in one pot solution by microwave-assisted extraction (MAE) and conventional extraction (CE) methods. MAE parameters were optimized to maximize yield and adjust in situ final physicochemical properties of extracted pectins, such as the methylation degree (DM), significantly influencing pectin functionality and application. Citric acid (CA) and acetic acid (Hac) were employed as solvents to mitigate pectin degradation. Extracted pectins were structurally (GPC and FTIR-ATR), morphologically (SEM), and thermally (TGA) characterized. From the reaction batch, the bioactive compounds (AOs) were separated and recovered, and their yield and antioxidant activities were evaluated with a DPPH assay. Moreover, by strategically selecting pH and solvents, this research enabled precise control over the final properties of pectin. The various characterization techniques employed show that the extraction conditions significantly influence the physicochemical and morphological properties of the material. Molecular weight (Mw) values range from 218 kDa to 567 kDa, surface morphology varies from compact/aggregated structures to three-dimensional network-like formations, and the DM spans from 34% (low DM) to 83% (high DM). This highlights a novel approach for predicting and tailoring in situ characteristics of extracted pectin to meet specific application requirements.

## 1. Introduction

Citrus fruits are widely consumed for their rich flavor and health benefits, particularly their high vitamin C and antioxidant content, which support immunity and overall well-being [1]. Global citrus production reached approximately 47.6 million tons in 2019–2020, with oranges accounting for 61% of total output [2]. However, only 40–60% of citrus biomass is consumed, leaving significant waste—over 60 million tons annually—posing environmental hazards due to its high fermentable sugar content [3,4]. On the other side, citrus waste is increasingly recognized as a valuable source of bioactive compounds, essential oils, dietary fiber, and particularly pectin [5,6]. Pectin, a key polysaccharide in citrus residues, is widely used in biomedical, pharmaceutical, food, and agricultural applications due to its biocompatibility and biodegradability [7,8,9]. Usually, pectin can be extracted from the citrus peel using acids as extractive agents. These latter are highly effective both in breaking cell walls, inside which pectin is preserved and in increasing solubilization, improving pectin final recovery. Therefore, the experimental extraction conditions, such as temperature, time, solid-to-liquid ratio, type of acid and pH, should be carefully selected and tuned to achieve pectin with desired structural and performing properties, as well as specific quality and yield. 

Recently, Zannini et al. [10] used conventional extraction (CE) methods to extract pectin and polyphenolic compounds from citrus pomace wastes. They investigated different extraction parameters and selected the best conditions providing the highest yield of pectin and, at the same time, the best structural properties, such as high molecular weight. Alongside pectin extraction and recovery, the same authors were able to successfully retrieve the antioxidant compounds from the hydroalcoholic fraction. Furthermore, the long exposure to solvent and high temperatures could affect the final physicochemical properties of pectin, as well as the bioactivity of polyphenol compounds, without neglecting the drawbacks related to the wide use of both solvents and energy seriously affecting the costs of the whole extraction process. Thus, the rationale for this study stems precisely from these limitations. Unlike CE, microwave-assisted extraction (MAE) offers a more efficient, eco-friendly alternative by reducing extraction time, solvent consumption, and energy input while simultaneously enhancing the yield and quality of pectin and bioactive compounds [11]. This study, therefore, addresses this gap by investigating how MAE parameters can be tailored to achieve enhanced pectin and bioactive compound recovery while sustainably maintaining their desirable properties. However, we are fully aware of the experimental constraints associated with MAE, including the need to carefully optimize microwave power, exposure time, and solvent composition to achieve an optimal balance between extraction efficiency and product integrity. These factors must be critically evaluated when comparing MAE with conventional extraction methods to ensure that the advantages outweigh the potential limitations.

Microwave-assisted extraction (MAE) represents one of the most modern and efficient methods used to extract and recover polysaccharides and bioactive compounds from vegetable matrices [12,13]. MAE is based on non-ionizing waves working in frequencies ranging from 300 MHz up to 300 GHz, generating rapid and instantaneous volumetric heating and rupture of the vegetable cell walls, thus both inducing a rapid and efficient solvent diffusion into the damaged structural walls and promoting macromolecules and bioactive compounds transferring to the solvent medium. Several studies have focused attention on the advantages of MAE with respect to the conventional extractive methods used to exploit citrus pomace as a source of high-added-value products [14,15,16]. Reference studies have weighed the advantages of MAE technology in terms of mass yield increasing and extraction time and solvent consumption reduction whilst disfavoring the conventional extraction methods needing longer times and high temperatures to produce and transfer the same heat responsible for the breakdown of cell walls and recovery of high-added values molecules [17,18].

In this study, MAE parameters were precisely optimized to enable the simultaneous extraction and recovery of pectin and bioactive compounds within a single-step (one-pot) process. This approach not only enhances efficiency by reducing processing time, solvent usage, and energy consumption but also allows direct modulation of key pectin physicochemical properties in situ. In particular, we strategically controlled the degree of methylation (DM), a crucial functional property influencing pectin’s gelling and film-forming capabilities. By fine-tuning the extraction conditions, we aimed to predict and tailor the molecular, structural, and performance characteristics of pectin, along with the antioxidant activities of the recovered polyphenols. To our knowledge, this integrated methodology has not been previously explored, making it a novel and valuable contribution to sustainable biopolymer extraction. Aqueous solutions of organic acids, such as citric acid (CA) and acetic acid (HAc), were selected as solvents due to their milder impact on pectin integrity compared to mineral acids [19,20]. The extracted pectins were structurally (GPC, FTIR-ATR), morphologically (SEM), and thermally (TGA/DTG) characterized while their DM was assessed. Additionally, polyphenols were recovered and evaluated for antioxidant activity using the DPPH assay. Pectin and antioxidants derived from citrus biomass present promising applications aligned with circular economy principles, minimizing waste while maximizing resource value. Their recombination enables the development of bioactive food packaging and edible coatings [7,21], further enhancing sustainability in material applications, agriculture [9], wound healing, nutraceutical, pharmaceutical and cosmetic products [22,23,24].

## 2. Materials and Methods

### 2.1. Material and Chemicals

The citrus waste used for the extraction was supplied by Università Mediterranea of Reggio Calabria (Italy). The initial moisture content was approximately 15% by weight and was removed through lyophilization. This process involved freezing the samples at −20 °C, then placing them in a lyophilizer set to −80 °C (cooling), −10 °C (plate temperature), and 0.031 mBar (vacuum) until a constant weight was achieved. The final moisture content was verified to be below 3% using thermogravimetric analysis (TGA) with the following parameters: a heating rate of 10 °C/min from ambient temperature to 120 °C, followed by a constant temperature of 120 °C for 20 min. Prior to being used, the freeze-dried citrus waste was ground in a blender to obtain a uniform particle size of 5–7 mm. Glacial acetic acid (HAc), citric acid (CA), isopropanol, sodium hydroxide (NaOH), hydrochloric acid (HCl), phenolphthalein and ethanol were purchased by Sigma-Aldrich, Milano, Italy. 1,1-Diphenyl-2-picrylhydrazyl (DPPH) used for antioxidant activity assay was provided from TCI (Tokyo Chemical Industry); citrus pectin “Aglupectin LC-S12P”, DM = 38%, was supplied by Silva Team, Cuneo, Italy.

### 2.2. Procedure of Pectin Extraction: Conventional Extraction (CE) and Microwave-Assisted Extraction (MAE)

The conventional experimental apparatus and extraction procedures in HAc were previously reported by Zannini et al. [10] and used to perform CA extraction. Briefly, citric acid extraction of pectin was performed as follows: 20 g of citrus waste powder was dispersed in 200 mL of CA water solution at pH = 2. The solution was heated at 100 °C for 6 h under stirring. In this experimental condition, pectin was easily released from the cell walls and solubilized in the acid solution, whereas the insoluble fraction was recovered and separated after filtration. Subsequently, the filtrate was centrifuged for 40–50 min at 10,000 rpm at 4 °C. The cooled solution proceeded to precipitation by the addition of two to four volumes of alcohol (isopropanol), followed by washing with ethanol to remove alcohol-soluble compounds and was put in an oven at 60 °C under dynamic vacuum (approximately 300 mBar) overnight. Pectin yield (PY) was calculated using the following equation:

(1) $$Pectin\;yield\left(\%\right)=\frac{mp}{m}\;*\;100$$

where *mp* is the weight of recovered dried pectin (g), and *m* is the weight of dried citrus waste powder (g). In order to recover the bioactive compounds from the residue in the hydroalcoholic fraction, the latter was evaporated through a rotary evaporator and the solid residue was recovered.

Based on previous findings [25], MAE of pectin was performed according to the following procedure: in a single PTFE vessel, within a 16-unit microwave apparatus, 1 g of citrus waste was placed and mixed with acidified water (HAc or CA) at pH levels of 2, 2.5, and 3 (5500 mM, 550 mM, 50 mM for HAc and 140 mM, 14 mM, 1.4 mM for CA respectively) which were directly prepared using a Titrando 888 Metrohm pHmeter in a solid-to-liquid ratio (SLR) of 1:20 and then exposed to microwaves action in a Multiwave5000 (Anton Paar, Graz, Austria) at 90 °C for 180 and at a microwave power of 422 Watts (W). As described above, after each extraction procedure, pectin was separated from the mixture by centrifugation (30 min at 10,000 rpm and 4 °C), followed by the precipitation of the supernatant with isopropanol in a 1:2 ratio (*v*/*v*). Finally, wet pectin was purified by consecutive washing with ethanol and dried up to a constant weight at a temperature of 60 °C.

## 3. Physico-Chemical Characterizations

### 3.1. Pectin Methylation Degree (DM)

The pectin methylation degree was determined by the titration method, as reported by Zannini et al. [10]. Briefly, 2 mL of ethanol and 20 mL of distilled water were added to 200 mg of pectin, and the mixture was kept at 60 °C under magnetic stirring until it reached complete dissolution. Subsequently, after adding two drops of phenolphthalein reagent to the mixture, the titration was carried out with a known volume of NaOH solution (0.1 M, *V*_1_) until the mixture color turned pink. Afterward, 10 mL of NaOH (0.1 M) was added to the solution and kept under stirring for 1 h at room temperature. In the next step, 10 mL of HCl (0.1 M) was added to the mixture and stirred up for clarification. Finally, the mixture was titrated again with NaOH solution (*V*_2_). DM percentage was estimated using the following Equation (2):

(2) $$DM\left(\%\right)=\frac{V_{2}}{V_{2}+V_{1}}\;*\;100$$

### 3.2. Radical Scavenging Activity Assays of Extracted Polyphenols by Means of 2,2-Diphenyl-1-picryl-hydrazyl-hydrate (DPPH)

Hydroalcoholic extract stock solutions (1.0 mg/mL) were prepared using the mass recovered from the lyophilization of hydroalcoholic solutions of pectin purification. These stock solutions were then diluted in ethanol to a final concentration of 800 µg/mL. Afterward, 1 mL of DPPH ethanol solution (0.1 mM) was added to 1 mL of sample solutions. The mixture was vortexed and taken at room temperature in the dark for 30 min. Then, the absorbance at 517 nm [26] was measured and the percentage of inhibition of the DPPH radical (DPPH according to the following Formula (3):

(3) $$\%\;\mathrm{of}\;\mathrm{inhibition}\;\mathrm{of}\;\mathrm{the}\;\mathrm{DPPH}\;\mathrm{radical}=\left[\frac{{Abs}_{control}-\left({Abs}_{sample}-{Abs}_{blank}\right)}{{Abs}_{control}}\right]\;*\;100$$

Ethanol (1.0 mL) was used as a blank, while DPPH ethanol solution (1.0 mL; 0.1 mM) and ascorbic acid were used as a negative and positive control, respectively.

### 3.3. Gel Permeation Chromatography (GPC)

Gel permeation chromatography was employed to determine the molecular weights of extracted pectins. Measurements were performed using a GPC Max Viscotek equipped with a TDA 305 triple detector array composed of a refractive index (RI), low-angle light scattering (LALS), right-angle light scattering (RALS), and viscometer (IV) detectors. The column set consisted of a pre-column TSK PWXL and TSK Gel GMPWXL. All samples were dissolved up to a concentration of ≅3 mg/mL and eluted in Milli-Q water containing 0.2% NaN_3_ and 0.1 M NaNO_3_. After complete dissolution, samples were filtered through a 0.22 μm CA filter. The injection volume was 100 μL, and the flow rate was 0.8 mL/min. The chosen method of analysis was triple point, calibrated with a PEO standard provided by Viscotek and with a narrow molecular weight distribution. The measurements, performed in duplicate at 40 °C, according to the temperatures of the columns and detectors, ran for 45 min.

### 3.4. Attenuated Total Reflection Fourier Transform Infrared Spectroscopy (FTIR-ATR)

Attenuated Total Reflection Fourier Transform Infrared (FTIR-ATR) spectroscopy was carried out on the samples in the form of powders. The spectra were collected on a Perkin-Elmer Spectrum 100 spectrometer (Waltham, MA, USA) equipped with a Universal ATR diamond crystal sampling accessory. All samples were analyzed at room temperature in the range 4000–650 cm^−1^, recorded as an average of 16 scans with a resolution of 4 cm^−1^. Before testing, all samples were dried in an oven at 60 °C for 24 h.

### 3.5. Thermogravimetric Analysis (TGA)

Thermogravimetric analyses of the extracted pectins were performed by using a thermogravimetric analyzer, TGA/DTA Perkin-Elmer Pyris Diamond, equipped with a gas station. About 3–4 mg of the sample was placed in an open ceramic crucible and heated from 25 °C up to 600 °C at a speed rate of 10 °C/min, under a nitrogen flow of 30 mL/min. Each measurement was preceded by an empty pan run, which was subtracted from each thermogram to correct instrumental drift. Before testing, all samples were dried in an oven at 60 °C for 24 h.

### 3.6. Scanning Electron Microscope (SEM)

Morphological analysis of pectin powder samples was performed by means of a scanning electron microscope (SEM) (Quanta 200 FEG, 338 FEI, Eindhoven, The Netherlands). Before analysis, surfaces were sputter-coated with a homogeneous layer (18 ± 0.2 nm) of Au and Pd alloy by means of a splattering device (MED 020, Bal-Tec AG, Tucson, AZ, USA). The micrographs were performed at room temperature and voltage of 20 kV in high vacuum mode. Before testing, all samples were dried in an oven at 60 °C for 24 h. For each sample, at least four micrographs were performed at different magnifications; the authors selected micrographs to provide information related to the structures of pectin extracted.

## 4. Results and Discussion

### 4.1. Comparison Between CE and MAE Methodologies: A Path to In Situ Pectin Properties Control

Finalized to provide an experimental set-up useful to reliably predict, reproduce and tune the properties of pectins and bioactive compounds, the authors performed several extraction attempts by means of CE and MAE in order to obtain reproducible experimental parameters for the “one-pot” achievement of pectin and bioactive compounds with specific and tailored properties. To this aim, two acids, i.e., acetic acid (HAc) and citric acid (CA), at different pH levels (2, 2.5, 3), temperatures (MAE T = 90 °C, CE T = 100 °C), and Solid–Liquid Ratios (SLRs) (MAE 1:20 g/mL, CE 1:10 g/mL) were used. However, the parameter considered in both methodologies is the extraction time, which was markedly different. Specifically, the CE method took six hours, while extraction by MAE took only 180 s. 

Thus, based on the entire experimental dataset, different pectin yields (PYs), bioactive compound yields (AOs), tuned molecular weights (Mws, Mns) and pectin Methylation Degrees (DMs) were obtained. Firstly, a comparison between all of the results from the CE and MAE methodologies is discussed. Table 1 details the parameters of CE and MAE extractions in terms of acids, pH, SLR, temperature, extraction time used, and related data of PY, the amount of the antioxidant (AOs), methylation degree (DM) and DPPH. Specifically, the HAc and CA acids were finalized to find the suitable experimental parameters of extraction and were used at different pH values of 2, 2.5, and 3; the corresponding identification codes for all samples have been indicated (PEC1 HAc, PEC1 CA for pH = 2, PEC2 HAc, PEC2 CA for pH = 2.5, PEC3 HAc, PEC3 CA for pH = 3). The samples extracted by the CE method have been coded as PEC4 Hac and PEC4 CA. Firstly, it is worth noting that the substantial difference between CE and MAE is mainly due to the following experimental parameters: extraction time, the energy required to break the cell walls of the citrus wastes, and the maximum yield recovered of pectin and bioactive molecules. From Table 1, it is possible to note that for MAE extraction, the maximum pectin yield was 22.5% by weight at pH = 2, SLR = 1:20 g/mL, T = 90 °C and extraction time of 180 s. In contrast, by exploiting the traditional extraction method (CE), six hours and T = 100 °C were necessary for obtaining slightly higher values of PY, AOs and DPPH.

CE relies on prolonged heating to facilitate solvent diffusion into plant cell walls, effectively breaking down the pectin-rich middle lamellae and enhancing extraction efficiency over time [27,28]. In contrast, MAE rapidly heats cell moisture through microwave radiation, enabling fast cell disruption and pectin release, which requires as little as 180 s in this study [29,30]. However, its rapid nature may limit solvent penetration, potentially reducing yield [31]. Despite slightly lower PYs, AOs, and DPPH values, MAE’s ability to co-extract pectin and bioactive compounds efficiently in a short time with lower energy consumption makes it a promising approach for sustainable industrial processing. In our system, MAE achieved near-total pectin extraction within three minutes. In addition, Table 1 data evidence that, by using CA and low pH, higher PY (23 wt.%) and polyphenolic-flavonoids molecules (AOs), ranging 15–20 mg/mL, and with lower DMs (30–40%) were detected compared to the use of HAc and higher pH. Pectin extraction is significantly influenced by the acidity of the extraction environment, as acidic conditions facilitate the hydrolysis of complex polysaccharide bonds within the plant cell wall lamella. Specifically, the protonation of carboxyl groups in pectin reduces the electrostatic repulsion between pectin molecules, thereby enhancing their solubility and extraction efficiency [32]. Citric acid provides a more favorable acidic environment due to its ability to maintain a low pH through multiple acidic sites, which is crucial for effective pectin solubilization. Additionally, the structural characteristics of pectin, such as molecular weight and viscosity, can be significantly affected by the type of acid used during extraction. Citric acid not only improves extraction efficiency but also contributes to the preservation of the functional properties of pectin, making it a preferred choice in many extraction protocols [33]. In contrast, acetic acid has a higher pKa (approximately 4.8) compared to the first dissociation constant of citric acid (pKa ≈ 3.1), meaning that citric acid more readily donates protons in solution, resulting in a lower pH under comparable conditions. This greater ability to acidify the medium makes citric acid more effective for pectin extraction, as the optimal pH range for this process is better maintained [34].

In terms of DM, the quality of extracted pectin can be assessed by the percentage of DM, which is an index of thickening and gelling properties that are absolutely fundamental to drawing up specific pectin applications in several industrial areas of food and pharmaceuticals. In agreement with Fraeye et al. [35], DM decreases proportionally with pH due to acid-catalyzed demethoxylation [36].

Thus, in the presence of CA and at pH 2 and 3, it is possible to evidence a drastic drop in DM due to the stronger environmental acidity. Moreover, in contrast, at pH 3, very high values of DM are obtained regardless of the acid used; anyway, an inhibitory effect on the extraction efficiency of both polysaccharides and bioactive molecules could be observed, highlighting that this pH significantly deviates from achieving optimal extractive efficiency [37]. To assess if some fractions of pectin and polyphenol remained in the solid residue of citrus biomass after the first extraction, a second MAE extraction on the same crude solid residue was performed under optimal conditions (pH 2, 90 °C, SLR 1:20, for 180 s). In Table 1, as an example, PEC1-CA-residue extraction yields were only 6.5 wt.% of pectin and 25 mg/mL of antioxidant compounds. These results demonstrated that a single three-minute MAE run was sufficient to extract nearly all the pectin from the starting citrus biomass, underscoring the validity, effectiveness and sustainability of the MAE method. Consequently, no further extraction attempts or prolonged trials were necessary from the same starting citrus biomass. In addition, it should be emphasized that modulating the initial MAE extraction parameters makes it possible to influence the structural characteristics of the extracted pectin, such as molecular weight and degree of esterification. While further studies are needed to comprehensively establish the extent of this modulation, our results indicate that process conditions play a role in defining pectin's final properties. Additionally, compared to the conventional extraction process (CE), pectins obtained through microwave extraction exhibited a lower degree of methoxylation and a higher molecular weight (Mw), as discussed in the following sections.

### 4.2. DPPH Assay

The antioxidant activity of the hydroalcoholic phase obtained during the purification of pectin was evaluated. The DPPH radical-scavenging activity of the hydroalcoholic extracts is shown in Table 1. According to Costanzo et al. [38], phenolic compounds in citrus fruits tend to exhibit higher radical-scavenging capacity at low pH. This could be due to the enhanced stability and reactivity of antioxidant compounds under acidic conditions, which may facilitate their ability to donate hydrogen atoms and interact with DPPH radicals, contributing to antioxidant activity. The highest DPPH radical-scavenging activity was observed for the extracts obtained using MAE with citric acid (CA) as the solvent. This could be related to the presence of functional hydroxyl groups, which may remain in their non-ionized form at low pH, potentially enhancing their ability to donate hydrogen atoms to neutralize free radicals like DPPH. Additionally, in a hydroalcoholic medium at low pH, the solubility and interaction of specific antioxidant compounds may improve, possibly supporting their interaction with DPPH radicals. Finally, the acidic environment may help preserve the structural integrity of certain antioxidants, which can be sensitive to degradation or oxidation at higher pH levels (as seen in Table 1, where lower scavenging activity is observed at pH = 3), thereby potentially contributing to increased DPPH scavenging activity.

### 4.3. Gel Permeation Chromatography (GPC)

**Figure 1:**
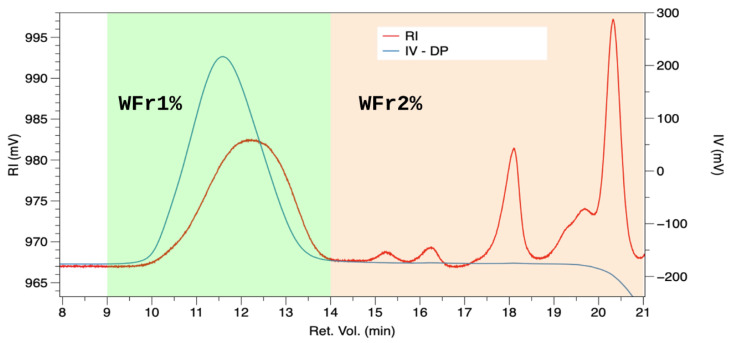
Representative GPC chromatogram superimposing the Refractive Index (RI in red) and Intrinsic Viscosity (IV in blue) of extracted sample PEC1-CA; the region in green is WFr_1_%, while in orange, WFr_2_%.

In Table 2, the weight-average molecular weight (Mw) and number-average molecular weight (Mn) are reported. In addition, WFr_1_% and WFr_2_% of analyzed samples are reported too. Specifically, WFr_1_% is the “effective” polymeric fraction, identified as part of the injected sample, showing signals at refractive index (RI) and intrinsic viscosity (IV) detectors at lower retention volume, while WFr_2_% is attributed to non-polymeric fraction, i.e., oligomers, monomers, molecules etc. showing signal only at RI at higher retention volume. In Figure 1, by way of clarifying, for example, GPC chromatograms of sample PEC1-CA detailing both RI and IV are reported; the region in green is WFr_1_% while in orange, WFr_2_%.

Previously, in Section 4.1 and Table 1, the overall trend suggested that by decreasing the pH, the yields increased. In terms of molecular weights, the decrease in pH generally leads to an increase in molecular weights up to pH 2.5. At a lower pH, yield further increased whilst polymer depolymerization in terms of molecular weights was observed. In particular, for the MAE samples, polymer degradation was mitigated by a very low extraction time, whereas samples extracted by the CE method evidenced a stronger decrease in Mw and WFr_1_, especially when citric acid was employed. GPC analysis of the samples indicated a significant variation in molecular weights between microwave-assisted extraction (MAE) and conventional extraction (CE), mainly due to the substantial difference in extraction times combined with the distinct nature of each technique. Notably, concerning the CE method, it is worth observing that the PEC4-CA sample is more degraded than PEC4-HAc, as pointed out by its dramatically decreased molecular weight fractions (WFr_1_%) in favor of a substantial increase of low molecular weights segments. This outcome suggests that the stronger nature of the acid used, coupled with the prolonged extraction time inherent in CE, accelerates degradation and the breakdown of the pectin structure into smaller segments. With regards to MAE samples, it is worth underlining that PEC1-CA results are more degraded than PEC1-HAc, as expected by the use of stronger acid, as widely discussed in Section 4.1 [39]. Moreover, the reduced Mw does not interfere with the envisaged performance of the pectin, for example, with its film-ability being an essential feature for pectin applications in several areas, as discussed in Section 4.7 regarding future perspectives. At pH 2.5, both sample (PEC2) yields dropped sharply (see Table 1), but they exhibited the highest molecular weights, along with comparable WFr_1_% and WFr_2_%; this could be due to the reduced extraction efficiency hindering the occurrence of depolymerization. Similar trends are observed with the PEC3 samples, wherein the low yield (see Table 1) was counterbalanced by high molecular weight and low macromolecular degradation. Finally, it could be hypothesized that, as a rule, a lower pH leads to increased yield. In terms of molecular weight, it appears that decreasing the pH results in higher molecular weights and improved extraction efficiency up to pH 2.5. At a lower pH, yield further increases with concurrent depolymerization mitigated by the low extraction time of MAE compared to CE, in particular when CA is employed.

### 4.4. Attenuated Total Reflection Fourier Transform Infrared Spectroscopy (FTIR-ATR)

**Figure 2:**
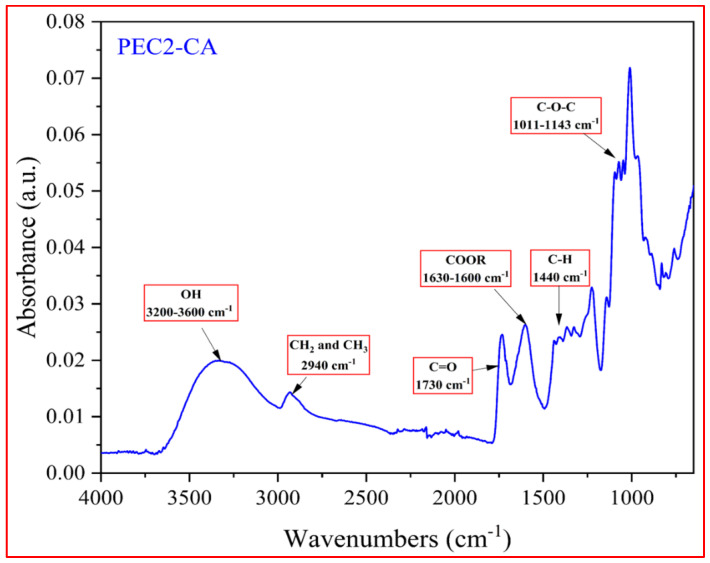
FTIR-ATR spectrum of the sample PEC2-CA.

In Figure 2, in order to better highlight the FTIR-ATR signals of pectin, the representative spectrum of the PEC2-CA sample is reported. All the pectin spectra have been postponed in the SM file, Figure S1. Observed spectra exhibit the same characteristic signals: the broad peak between 3200 and 3600 cm^−1^ is referred to OH groups while the one at 2940 cm^−1^ is related to CH_2_ and CH_3_ groups [40]. In addition, the peak at approximately 1730 cm^−1^ is attributed to the C=O ester. Carboxylate groups have two peaks: one peak is due to the asymmetrical vibrations at the range 1630–1600 cm^−1^, and another peak is due to weaker symmetric vibrations at 1440 cm^−1^. The two intense absorptions at 1011 and 1143 cm^−1^ are related to a glycosidic linkage between sugar units [41].

### 4.5. Thermogravimetric Analysis (TGA)

**Figure 3:**
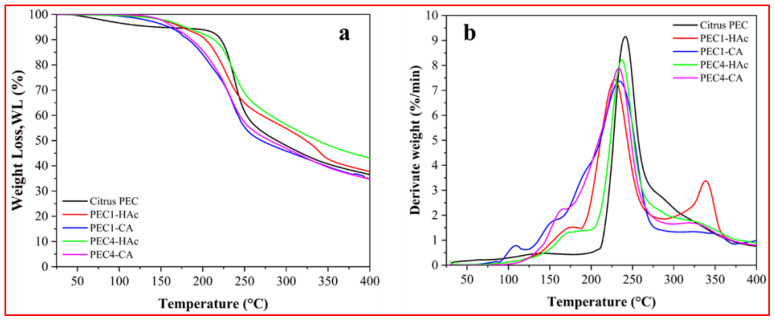
(**a**) TGA and (**b**) DTG thermograms of pectins extracted with CE (PEC4-Hac; PEC4-CA) and MAE (PEC1-HAc; PEC2-CA) methods.

Normalized TGA and the first derivate curves (DTG) thermograms are reported in Figure 3a,b, whereas the main thermal parameters are summarized in Table 3. To maintain this paper’s focus and better highlight the main differences in the thermal behavior of the samples, only the citrus pectin, PEC1, and PEC4 samples are discussed. In addition, to emphasize the main thermal difference among the samples, the TGA and DTG thermograms have been magnified in the 25–400 °C range. In the SM file, the TGA and DTG thermograms of all the samples are reported (Figure S2).

The thermal parameters investigated were the onset of temperature degradation (Tonset) taken as the temperature at which a 10% weight loss (WL) of samples occurred, and the temperature of the peaks of the DTG curves corresponding to the temperature of maximum degradation rate (Tpeak).

From the analysis of the TGA thermograms (Figure 3a), we can observe that the thermal degradation pattern of extracted pectins is very similar [29]. In general, the pectins obtained by the MAE and CE process showed three main thermal steps: the first ranged between room temperature, and approximately 120 °C was ascribed to the free and bound water evolution; the second step, up to 180 °C, was mainly due to the removal of low molecular weight molecules as the residual antioxidant fractions. This hypothesis is supported by the observation that, in this region, the commercial pectin (black curve) exhibited no weight loss, DTG peak, or shoulder, which is consistent with the absence of bioactive compounds. 

Furthermore, the DTG curves and T_peak_ values confirmed that the Citrus PEC was the most thermally stable sample, as expected due to its commercial purity. In the temperature range of 200–300 °C, fast kinetics of weight loss (50 %wt) occurred in all samples; they were associated with the thermal decomposition of the pectin. It should be highlighted that PEC1-CA and PEC4-CA have a more complex degradative profile in this region, mainly evidenced by DTG curves. It is likely that, independently from the extraction method, citric acid could push towards deeper degradation of more thermally susceptible low molecular weight polymer fractions. This experimental result matches the relatively low molecular weights evidenced by GPC analysis, as previously discussed. Finally, in the third thermal step (400–600 °C), slow weight loss was observed due to the decomposition of the residual lignocellulosic fraction [42]. DTG analysis of all the samples evidenced the presence of small broadened peaks at around 350 °C, likely due to the presence of cellulose fraction residues; it was possible to emphasize that CA was able to minimize hemicellulose and lignocellulose extraction, thus improving final pectin quality [43,44].

### 4.6. Scanning Electron Microscopy (SEM)

To investigate the impact of different extraction methods (CE and MAE) on pectin structural morphology, SEM micrographs were obtained for selected pectin samples. To maintain conciseness, we report and discuss representative micrographs of PEC4-HAc, PEC4-CA (CE-extracted pectin), and PEC1-HAc, PEC1-CA (MAE-extracted pectin).

**Figure 4:**
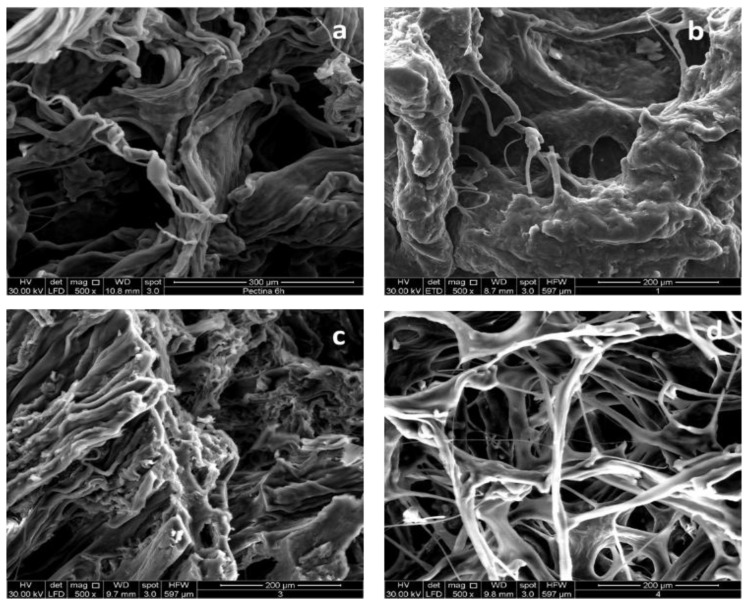
SEM micrographs of PEC4-HAc (**a**) and PEC1-HAc (**b**), PEC4-CA (**c**) and PEC1-CA (**d**) samples.

A previous study by the same authors [10] indicated that CE-extracted pectin with HAc solvent exhibited irregular intercellular spaces with rough and uneven surface characteristics. In line with this, the PEC4-HAc sample (Figure 4a) displayed a compact and aggregated morphology with irregular surfaces and a denser appearance. The presence of partially isolated microfibrils interspersed with dark voids suggests a less porous structure potentially influenced by the limited solubilization capacity of acetic acid and the prolonged CE process, which may promote molecular interactions and structural stabilization. For PEC1-HAc (Figure 4b), a smoother and more swollen surface was observed, with a denser morphology and fewer visible voids. This structural appearance aligns with the rapid heating and cell wall breakdown associated with microwave-assisted extraction [45]. However, some degree of aggregation remains, suggesting that acetic acid may be less effective in creating a more defined structure compared to citric acid. Regarding the PEC4-CA sample (Figure 4c), a more disordered, smoother, and less compact morphology was evident. The greater acidic strength, solubility, and chelating ability of citric acid likely contributed to enhanced breakdown of plant cell walls, resulting in a more fibrous and open structure with interwoven microfibrils [46]. However, the conventional extraction method may still limit complete structural disintegration. Finally, the PEC1-CA sample (Figure 4d) obtained through MAE exhibited the most open and porous morphology, forming a three-dimensional network-like structure. The synergistic effect of microwave energy and the acidic environment provided by citric acid facilitated cell wall disruption, yielding a highly porous pectin structure. This characteristic may enhance pectin’s functional properties, particularly for applications requiring improved gelling ability and molecular interactions [47].

### 4.7. Future Outlooks

In this study, the authors concentrated on improving MAE extraction methods to obtain pectin and antioxidants through sustainable, eco-friendly processes that effectively modulate their properties in situ. The potential applications of these extracted and characterized compounds will be explored in a forthcoming publication. 

**Figure 5:**
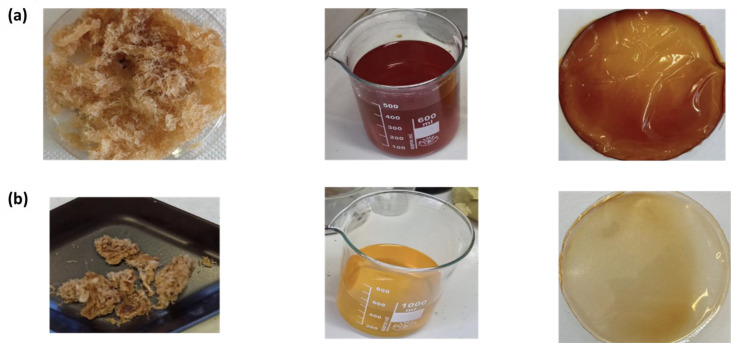
(**a**) PEC1-CA MAE Pectin, antioxidant hydroalcoholic solutions, and their recombination in precasted film; (**b**) PEC1-HAc MAE Pectin, antioxidant hydroalcoholic solutions, and their recombination in precasted film.

To provide clear and compelling evidence of the film-forming ability of pectins and the development of bioactive films by the inclusion of antioxidants in the polymer matrix, as an example, the authors provided photos in Figure 5 of the extracted and dehydrated pectin, hydroalcoholic antioxidant solutions, and solvent-cast films resulting from their physical recombination. This novel approach represents a promising pathway for sustainable applications, including functional food packaging, edible coatings, and bioactive biomedical films [48]. Moving forward, the authors will focus on optimizing the extraction processes to maximize yield and quality while preserving eco-energy-efficient protocols. In addition, an in-depth cost analysis of the process will be performed in order to assess the most cost-effective approach to be exploited. Embracing a circular economy model, the integrated approach to recovering and upgrading citrus wastes supports sustainability and fosters the development of eco-friendly products that meet industry standards for functionality and environmental stewardship, in this way reducing the lacuna between laboratory research and commercial applications.

## 5. Conclusions

In conclusion, MAE and CE methods have been used in this study to extract both pectin and antioxidant molecules from citrus waste. While the extraction yield from MAE was found to be slightly lower than that obtained through the CE method, the significant advantages of MAE, such as its drastically reduced extraction time and environmentally friendly nature, make it the more sustainable and energy-efficient extraction path. Furthermore, this research demonstrated that by carefully choosing the pH and type of solvent (e.g., acetic acid or citric acid), it is possible to in situ tailor the final physicochemical properties of the extracted pectin, including its degree of methylation (DM) and molecular weight, to suit specific applications, such as optimizing its gelling behavior, a feature widely exploited in the quite whole pectin applications. These findings highlight the potential of fine-tuning extraction conditions to enhance the functionality of pectin and antioxidants for various industrial uses, thereby both promoting innovative and sustainable practices in the valorization of citrus wastes and fostering sustainable practices by maximizing natural resource efficiency and promoting innovative utilization of natural biodegradable sources.

## Figures and Tables

**Table 1:** Experimental parameters (pH, SLR, T, and t) for pectin (PY) and bioactive compounds (AOs) extraction based on MAE and CE methods. All MAE data were analyzed, including over 16 replicas of the microwave apparatus, while CE data was noted in triplicate.

MAE	pH	SLR (g/mL)	Temperature (°C)	Time (s)	PY (%)	AOs (mg/mL)	DM (%)	DPPH (%)
PEC1-HAc PEC1-CA	2	1:20	90	180	10.6 ± 1.5 22.5 ± 1.3	6.4 ± 1.8 19.8 ± 1.4	49.0 ± 3.0 34.0 ± 2.5	10.6 ± 1.0 10.5 ± 1.3
PEC2-HAc PEC2-CA	2.5	1:20	90	180	10.8 ± 0.9 9.5 ± 1.1	3.3 ± 1.0 7.2 ± 0.5	62.1 ± 1.7 43.6 ± 2.8	4.1 ± 0.8 7.0 ± 1.4
PEC3-HAc PEC3-CA	3	1:20	90	180	7.7 ± 1.4 7.3 ± 1.1	4.7 ± 1.2 5.8 ± 1.3	83.5 ± 3.2 72.7 ± 1.1	1.2 ± 0.5 5.6 ± 2.1
PEC1-CA-residue	2	1:20	90	180	6.5 ± 1.0	25.2 ± 3.2	16.5 ± 2.4	5.4 ± 2.6
** CE **	**pH**	**SLR (g/mL)**	**Temperature (°C)**	**Time (h)**	**PY (%)**	**AOs (mg/mL)**	**DM (%)**	**DPPH (%)**
PEC4-HAc	2	1:10	100	6	24.1 ± 2.2	16.5 ± 1.7	70.3 ± 1.3	6.1 ± 2.2
PEC4-CA	2	1:10	100	6	30.0 ± 2.7	20.7 ± 2.0	28.6 ± 1.8	9.8 ± 1.3

**Table 2:** Weight-average molecular weight (Mw) and number-average molecular weight (Mn), effective polymeric fractions WFr_1_%, and WFr_2_% of extracted pectins.

Samples	M_n_ (kDa)	M_w_ (kDa)	WFr_1_%	WFr_2_%
PEC1-HAc	195.575	390.166	74	26
PEC1-CA	134.352	218.951	53	47
PEC2-HAc	233.713	472.280	54	46
PEC2-CA	294.180	567.688	55	45
PEC3-HAc	186.208	377.988	59	41
PEC3-CA	142.157	231.053	64	36
PEC4-HAc	43.775	118.463	63	37
PEC4-CA	35.303	93.269	33	67

**Table 3:** Thermal parameters of extracted pectins by MAE and CE methods; onset of temperature degradation (T_onset_), and the temperature of peaks corresponding to the temperature of the maximum degradation rate (T_peak_).

Samples	T_onset, 10% wt._ (°C)	T_peak1_ (°C)	T_peak2_ (°C)	T_peak3_ (°C)
Citrus PEC	221.5	126	241.6	-
PEC1-HAc	203.3	174.3	228.3	338.6
PEC1-CA	182.6	154.1	234.3	336.2
PEC4-HAc	213.1	171.2	236.9	333.1
PEC4-CA	186.2	165.2	232.9	327.0

## Data Availability

The original contributions presented in this study are included in the article/supplementary material. Further inquiries can be directed to the corresponding author.

## Supplementary Materials

The following supporting information can be downloaded at: https://www.mdpi.com/article/10.3390/polym17050659/s1, Figure S1: FTIR-ATR of commercial pectin and all pectins extracted with MAE and CE methods; Figure S2: (a) TGA and (b) DTG thermograms of commercial and extracted pectins.
