# Supplementary material for: Citrus Wastes as Source of Pectin and Bioactive Compounds Extracted via One-Pot Microwave Process: An In Situ Path to Modulated Property Control
Source: Polymers (Basel). 2025 Feb 28;17(5):659. doi: 10.3390/polym17050659 (PMC11902335; doi:10.3390/polym17050659)
Supplement: Supplementary file 1 [file polymers-17-00659-s001.zip › polymers-3481583-supplementary.pdf]

Supplementary Materials

Citrus Wastes as Source of Pectin and Bioactive Compounds Extracted via One-Pot Microwave Process: An In Situ Path to Modulated Property Control †

Domenico Zannini [1,‡], Martina Monteforte [2,‡], Luca Gargiulo [2], Tiziana Marino [2], Giovanna Gomez d'Ayala [2], Gabriella Santagata [2,*] and Giovanni Dal Poggetto [2]

[1] Institute of Chemical Sciences and Technologies "G. Natta" (SCITEC), National Council of Research, Via De Marini 6, 16149 Genova, Italy; domenico.zannini@cnr.it

[2] Institute for Polymers, Composites and Biomaterials (IPCB), National Council of Research, Via C. Flegrei 34, 80078 Pozzuoli, Italy; martinamonteforte15@gmail.com (M.M.); lucagargiulo@cnr.it (L.G.); tiziana.marino@cnr.it (T.M.); giovanna.gomezdayala@cnr.it (G.G.d.); giovanni.dalpoggetto@cnr.it (G.D.P.)

* Correspondence: gabriella.santagata@cnr.it; Tel.: +39-0818675214

† In memory of our dear scientist, mentor, friend and collegue Dr. Mario Malinconico.

‡ These authors contributed equally to this work.

Abstract: In this paper, citrus pomace was used as a source of pectin and polyphenols extracted in one pot solution by microwave-assisted extraction (MAE) and conventional extraction (CE) methods. MAE parameters were optimized to maximize the yield and tailor the final physicochemical properties of extracted pectins in situ, such as the methylation degree (DM), significantly influencing pectin functionality, including its gelling behavior, which is essential for pectin application. Citric acid (CA) and acetic acid (Hac) were employed as solvents to mitigate the pectin degradation typically observed using stronger mineral acids. The extracted pectins were structurally (GPC and FTIR-ATR), morphologically (SEM), and thermally (TGA) characterized, and their DM was also evaluated. The antioxidants (AOs) were also separated and recovered, and their yield and antioxidant activities were evaluated through DPPH assay. Moreover, by strategically selecting pH and solvents, this research enabled precise control over the final properties of pectin, showcasing a novel method to anticipate and achieve desired characteristics for its specific foreseen applications in situ. The overall results of this study evidenced that, although CE provides slightly higher pectin and polyphenol yields, the best results are obtained by using MAE, CA, and low pH to best match all of the properties in terms of AOs and pectin yields, as well as of structural, morphological and thermal properties of the pectins. In addition, the MAE method demonstrated substantial advantages in drastically reducing extraction time and energy, reinforcing its role within a circular economy framework, and showcasing its capacity to convert citrus pomace wastes into high-value products.

Results and Discussion

S.2.1. Attenuated Total Reflection Fourier Transform Infrared Spectroscopy (FTIR-ATR)

Figure S1 shows the FTIR-ATR spectra of commercial pectin and all pectins extracted with MAE and CE methods.

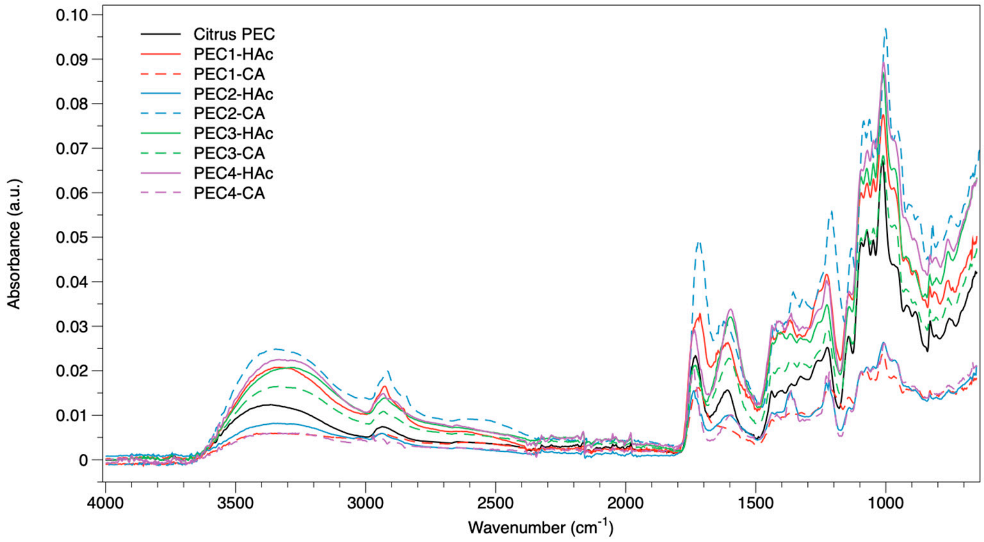

Figure S1. FTIR-ATR of commercial pectin and all pectins extracted with MAE and CE methods

S.2.2. Thermogravimetric Analysis (TGA)

Figure S2 shows the TGA (**Figure S2 a)** and DTG (**Figure S2 b)** thermograms of commercial and extracted pectins.

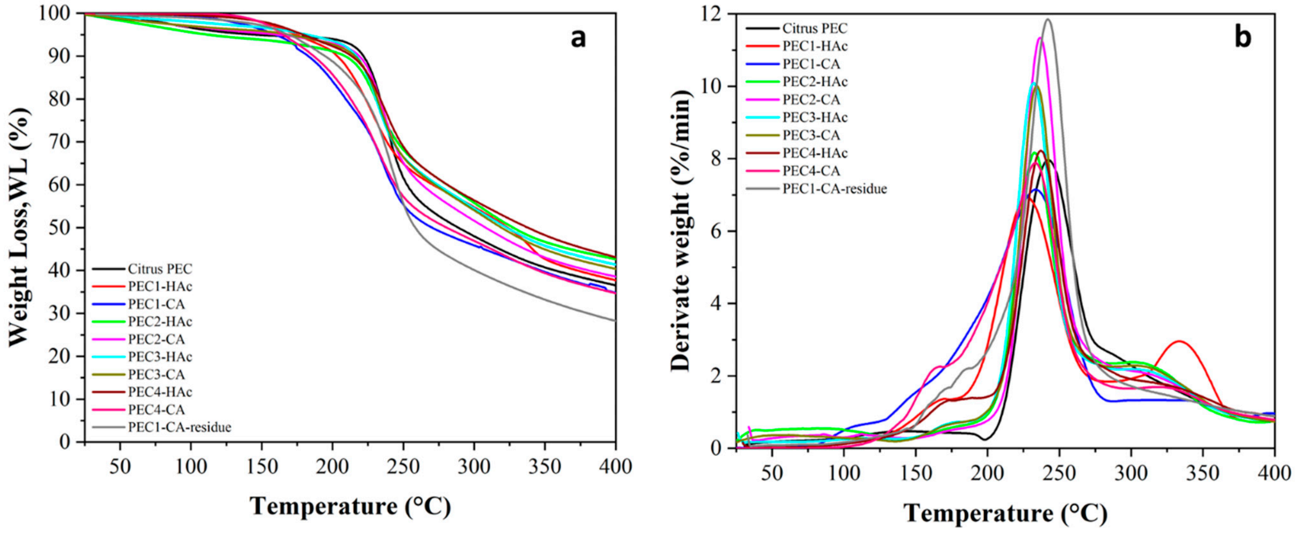

Figure S2. (a) TGA and (b) DTG thermograms of commercial and extracted pectins
